# Comprehensive evaluation of Medtronic’s Butterfly platform, a new audiovisual information material for patient education and shared decision-making in surgical thyroid disease

**DOI:** 10.1186/s12911-026-03526-w

**Published:** 2026-04-29

**Authors:** Maria Moschofidou, Grégoire Racine, Ilaria Giordani, Dionysios V. Chartoumpekis, Adelina Ameti, Gerasimos P. Sykiotis

**Affiliations:** Lausanne University Hospital and University of Lausanne, Lausanne, Switzerland

**Keywords:** Preoperative information, Patient education, Audiovisual information material, Thyroidectomy, e-Health, Shared decision-making

## Abstract

**Background:**

To facilitate shared decision-making, patients increasingly rely on online platforms for health-related information. While thyroid diseases are common, the quality of available thyroid-related information varies. This study comprehensively evaluated a new audiovisual information material developed by Medtronic for patients with surgical thyroid disease in English and French.

**Methods:**

Data were collected from patients at baseline (t1), after accessing the French version of Medtronic’s Butterfly platform but before surgery (t2), and three weeks post-surgery (t3). Patients assessed the material’s usefulness (USE), impact (eHIQ) and quality (Brief DISCERN), provided feedback on its content using a custom debriefing questionnaire, and self-reported on their anxiety (GAD-7), depression (PHQ-9) and stress (PSS-14). We assessed both versions for readability (FRES, FKGL, SMOG, GFI, and Scolarius); understandability and actionability (PEMAT-A/V); linguistic aspects (LIWC-22) and tone style (YesChat Tone Analyzer). In addition to quantitative and qualitative analyses of the respective datasets, triangulation was used to integrate both approaches.

**Results:**

Of 26 patients enrolled, 24 participated at t2 and 22 at t3; both benign and malignant/potentially malignant surgical indications were represented. The material’s usefulness and impact scores at t2 were significantly above respective predefined thresholds. Consistently, qualitative analysis showed that most participants found the material useful/ very useful. Whereas anxiety and depression scores were low across t1-t3, levels of stress were consistently high; PSS-14 scores at t1 correlated positively with eHIQ-Part 2 scores at t3 (*r* = 0.495, *p* = 0.019), suggesting that patients with higher stress may experience a greater impact. Even though readability scores for both languages indicated higher complexity than the generally recommended 6th -8th grade level, neither the quantitative nor the qualitative patient feedback indicated challenges with the material’s language. Assessment of the material by five investigators showed very good understandability and excellent actionability. Linguistic analysis showed a somewhat higher complexity of the French version; for both versions, tone analysis reported a clear, approachable, and professional style, with direct and informative content, using generally simple language. Finally, participants suggested slight improvements, especially regarding online navigation.

**Conclusions:**

These findings provide preliminary evidence that the Butterfly platform may serve as a useful educational resource for patients undergoing thyroid surgery; further validation in larger and more diverse populations is warranted.

**Supplementary Information:**

The online version contains supplementary material available at 10.1186/s12911-026-03526-w.

## Introduction

Despite efficient diagnostic tools and treatments for patients with thyroid diseases, their health-related quality of life (HRQoL) is often negatively impacted [1, 2]. Similarly, despite an excellent overall prognosis in the majority of cases, thyroid cancer survivors also experience a reduction in their HRQoL [3, 4]. Surgery is a critical moment in the trajectory of many patients with benign or malignant thyroid disease, because patient experience, surgical complications and other outcomes can have a major impact on HRQoL [5–7]. Thyroid surgery may be indicated for patients with thyroid cancer, large nodules that cause symptoms, nodules with a malignant, suspicious or indeterminate cytology [8], or conditions that cause hyperthyroidism [9]. Patient education is crucial in the decision-making of patients undergoing thyroid surgery to help them understand the procedure and its associated risks and benefits, potential alternatives to surgery, technical means to minimize complications, priorities in postoperative care, etc [7, 10]. This is consistent with studies in other medical fields showing that effective educational interventions can foster cooperation and improve postoperative outcomes [11]. In general, well-informed patients tend to experience less anxiety and more satisfaction with care, and they adhere better to postoperative instructions [11–14].

Online patient information materials help to shift the care model from physician-dominated to collaborative and patient-centered [15], thereby facilitating shared decision-making [13, 16, 17]. Challenges associated with such materials include their variable quality, reliability and accuracy, that may sometimes lead to misinformation or misunderstanding, as well as potential deviations from the information provided by healthcare providers (HCPs) that can lead to confusion or even conflict [18–20]. These general principles and concerns also apply to the online information on thyroid diseases, which is often incomplete, particularly on critical topics such as diagnosis and treatment [21]. For example, a study evaluating online information for the treatment of low-risk thyroid cancer indicated that most websites failed to incorporate updates from the 2015 American Thyroid Association (ATA) guidelines, and they often lacked essential details about treatment options necessary for informed decision-making [22]. Further, a review by the National Institute for Heath and Care Excellence (NICE) found high-quality evidence from 3 studies highlighting patients’ need for further information, and emphasized that the lack of sufficient information provided verbally by HCPs may lead patients to potential online misinformation [23].

The Medtronic Butterfly platform is an innovative e-learning tool designed to enhance education for patients undergoing thyroid surgery. It was developed in collaboration with a multidisciplinary group of surgeons, endocrinologists, and patient advocacy organizations. Its goal is to improve patients’ understanding of their thyroid conditions and the procedures they may undergo by offering (i) learning modules on thyroid conditions and treatments; (ii) step-by-step guides to surgical procedures and recovery expectations; (iii) insights into thyroid surgery; and (iv) post-surgery advice on managing recovery and adjusting to life after thyroid surgery. The co-construction process will be described in detail elsewhere. The platform’s main menu comprises five sections with written information: “Understanding your condition, diagnosis and treatment options”; “Getting ready for your hospitalization”; “Understanding your hospitalization and surgery”; “Next steps and advice for the recovery period”; and “Post-surgical treatments”. Additionally, the platform features five educational videos: “What is the thyroid?”; “Diagnostic process”, “Understand the surgery; Thyroid Surgery”; “Understand your hospitalization”; “Taking care of your scar”. To complement this information, three downloadable leaflets provide practical guidance: “What to pack for my hospital stay”; “Pre-admission checklists”; and “Recommendations for recovery”. The platform is freely accessible online in English (https://www.medtronic.com/content/dam/medtronic-com/xd-en/impact/elearning/thyroid-content-solution/story.html) the French version is in the process of being made available. The present study performed a comprehensive evaluation of the Medtronic Butterfly platform.

## Methods

### Study objectives, outcome measures, instruments, and schedule of assessments

The main objective of this single-arm, single-center cohort study was to perform a comprehensive evaluation of Medtronic’s Butterfly platform. The outcome measures, instruments, and schedule of assessments are indicated in Table 1. Sociodemographic and clinical data were collected at study enrolment (t0). Patient-assessed parameters were recorded before accessing the material (t1, 0–2 weeks after t0), after accessing the material but prior to surgery (t2, 0–2 weeks before surgery), and post-surgery (t3, 2–4 weeks after surgery). The duration between t1 and surgery varied, with a minimum interval of 2 weeks. At t1, patients completed questionnaires in person, with assistance from a doctoral student if necessary, and were then given access to the information material via a hyperlink. At t2 and t3, questionnaires were mailed to participants, who returned them using pre-paid envelopes.

**Table 1:** Outcome measures, instruments and schedule of assessments

Questionnaires	Variables	Evaluators	Time points			
			t0	t1	t2	t3
USE	Usefulness*	Patients			+	+
eHIQ^£^	Impact*	Patients			+	+
GAD-7	Anxiety^¶^	Patients		+	+	+
PHQ-9	Depression^¶^	Patients		+	+	+
PSS	Stress^¶^	Patients		+	+	+
Brief DISCERN	Quality^§^	Patients				+
Debriefing^£^	Content^§^	Patients				+
CRF-t1	Sociodemographic and clinical data	Investigators		+		
CRF-t2		Investigators			+	
CRF-t3		Investigators				+
FKGL, GFI, SMOG, FRES, Scolarius	Readability^§^	Investigators	+			
PEMAT-A/V	Understandability^§^	Investigators	+			
PEMAT-A/V	Actionability^§^	Investigators	+			
LIWC-22	Linguistic Analysis	Investigators	+			
YesChat Tone Analyzer	Tone Style Analysis	Investigators	+			

*main primary outcome measure; ^§^further primary outcome measure; ^¶^secondary outcome measure; 0: study enrolment; t1: before accessing the materials, 0–2 weeks after t0; t2: 0–2 weeks before surgery; t3: 2–4 weeks after surgery; USE: Usefulness Scale for Patient Information Material; eHIQ: e-Health Impact Questionnaire; GAD-7: Generalized Anxiety Disorder-7; PHQ-9: Patient Health Questionnaire-9; PSS: Perceived Stress Scale; CRF: case report form; FKGL: Flesch-Kincaid Grade Level; GFI: Gunning Fog Index; SMOG: Simple Measure of Gobbledygook; FRES: Flesch Reading Ease Score; PEMAT-A/V: Patient Education Materials Assessment Tool for Audiovisual Materials; LIWC-22: Linguistic Inquiry and Word Count; ^£^based on items developed from a literature review, and pilot-tested with the first 5 patients; cross-culturally validated French translation in strict adherence with the methodology recommended by Oxford University

### Study participants and sample size calculation

Eligible patients were adults who could understand and complete questionnaires in French, were able to use online resources via a computer, tablet or cell phone, and had an indication for thyroid surgery for either benign thyroid disease (Graves’ disease or benign nodules/goiter) or for suspected (indeterminate or suspicious cytology) or confirmed thyroid cancer. Patients were excluded if they were unable to follow procedures or provide consent or had had prior thyroid surgery. Patients were recruited at Lausanne University Hospital by proposing the study to all eligible patients between May 2022 and May 2023. The study was performed in accordance with the Declaration of Helsinki; it was approved by the Cantonal Commission on Ethics in Human Research of Vaud (Swiss Business Administration System for Ethics Committees identification number 2022 − 00395), and all patients provided written informed consent.

The target sample size was calculated for the main primary outcome of impact, hypothesizing a mean score of at least 5 points (medium effect size) above the 65-point threshold for good impact in Part 2 of the eHIQ questionnaire [24]. For a one-sample t-test, two-tailed significance p-value < 0.05 and statistical power of 80%, G*Power [25] calculates a sample size of 27 patients, yielding an actual power of 83.6%.

### Data handling and statistics

Data management was conducted using REDcap 14.0.12, and quantitative analyses were performed with GraphPad Prism 9.5.1 (GraphPad, San Diego, CA). For the main primary outcomes (eHIQ and USE scores), data were analyzed using one-sample t-test. For comparison of outcomes at different timepoints, data were analyzed using paired t-test (two timepoints; eHIQ and USE scores) or one-way repeated measures non-parametric ANOVA (three timepoints; GAD-7, PHQ-9 and PSS-14 scores). For comparisons between different patient groups, data were analyzed using paired t-test. For simplicity of presentation, data (eHIQ) were rounded to the nearest integer. Data on the material’s content (debriefing questionnaire) were analyzed qualitatively using Clarke and Braun’s six-step process for thematic analysis [26]. Data triangulation was achieved through integration of quantitative and qualitative data, including also linguistic aspects (LIWC-22, quantitative) and tone style (YesChat Tone Analyzer, generative artificial intelligence).

## Results

### Patient cohort

A total of 54 potential participants were initially contacted; after screening, 48 met the enrolment criteria, among whom 28 agreed to participate (58% acceptance rate). Two were excluded at t0 due to changes in their treatment plan; of the 26 participants, 22 completed the study (there were 2 dropouts before t2 and two before t3; 15% total dropout rate). The mean and median age of participants was 50 and 49 years, respectively (standard deviation 14.76, range 20–75); and 17 (65%) were female. Operations planned were total thyroidectomy (*n* = 12, 46%) and lobo-isthmectomy (*n* = 14, 54%). All types of surgical indications were represented: Graves’ disease (*n* = 3, 12%), benign thyroid nodule (*n* = 7, 27%), thyroid nodule with cytology results that were indeterminate (Bethesda III or IV) or suspicious for malignancy (Bethesda V) (*n* = 11, 42%), and thyroid cancer (*n* = 5, 19%). Nearly half (*n* = 12, 46%) of the patients reported that the disease was detected due to symptoms; in 5 patients (19%), diagnosis was secondary to detection of a neck lump, and in 9 (35%) it was an incidental finding on imaging. Only 5 participants (19%) had an education level below USA grade 9.

### High perceived usefulness and impact before surgery

**Fig. 1:**
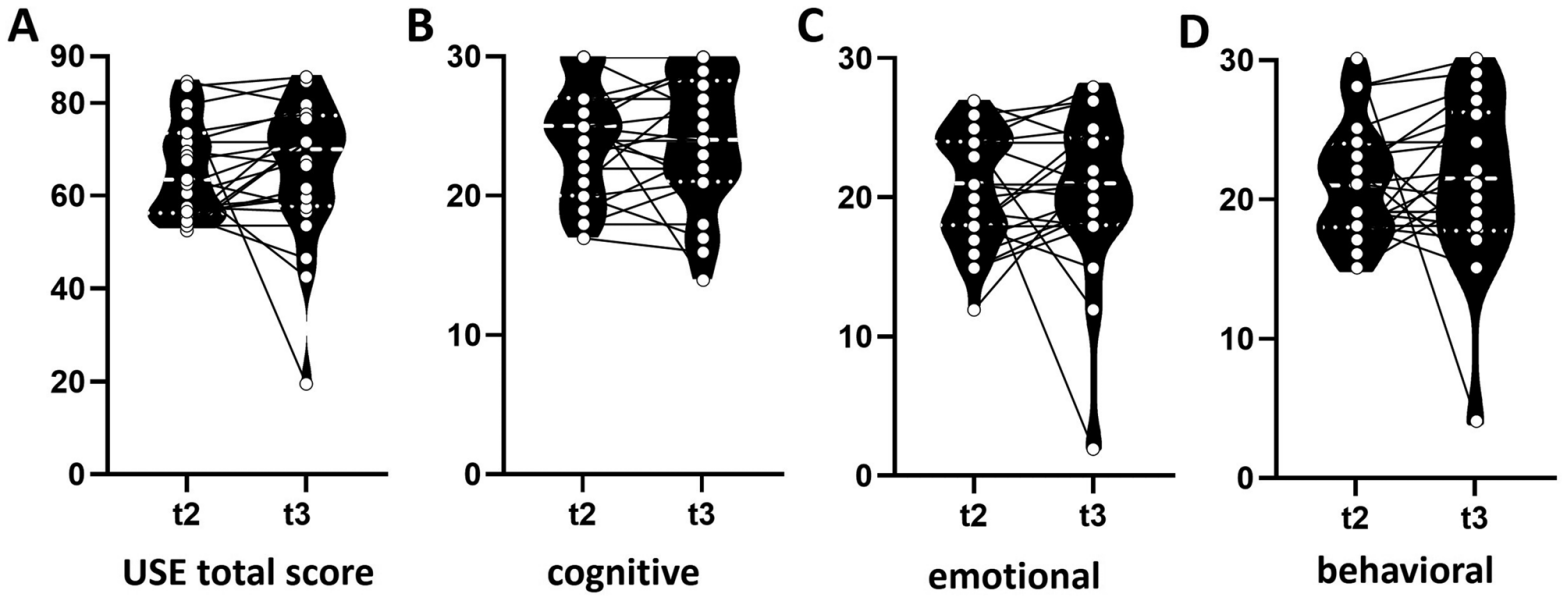
(**A**) Usefulness Scale for Patient Information Material (USE) total scores before (t2) and after (t3) surgery. (**B**-**D**) Respective subscale scores. The violin plots show individual patient data and indicate their respective medians and quartiles

USE results are shown in Fig. 1. Among the patients who completed the questionnaire at t2 (*n* = 24) and t3 (*n* = 22), the mean total score for usefulness was 66 ± 10 (range 53–85) at t2 and 66 ± 15 (range 20–86) at t3 (Fig. 1A), exceeding the predefined threshold of 60/90. Subscale results showed that the material supported patients cognitively, emotionally, and behaviorally, with patterns in the three subscales being similar to the total scores (Fig. 1B-D). Most patients scored in the upper half of the range, and there were no significant differences in scores between t2 and t3 in any of the subscales. Taken together, these findings indicate that the written part of the information material was useful for most patients before surgery, especially at the cognitive level, but also at the emotional and behavioral levels.

**Fig. 2:**
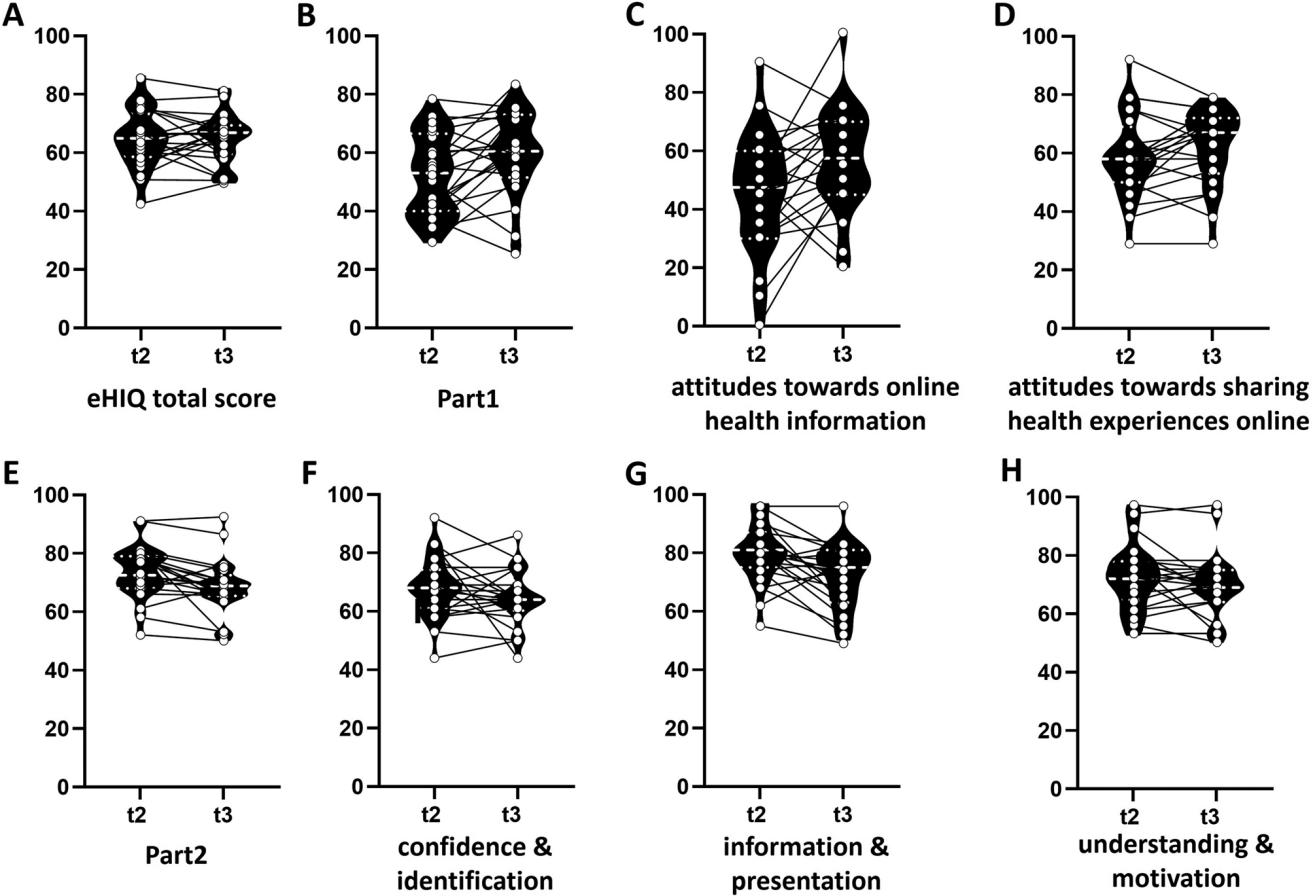
(**A**) e-Health Impact Questionnaire (eHIQ) total scores before (t2) and after (t3) surgery. (**B-D**) Respective scores of eHIQ-Part1 and its two subscales. (**E-H**) Respective scores of eHIQ-Part2 and its three subscales. The violin plots show individual patient data and indicate their respective medians and quartiles

**Fig. 3:**
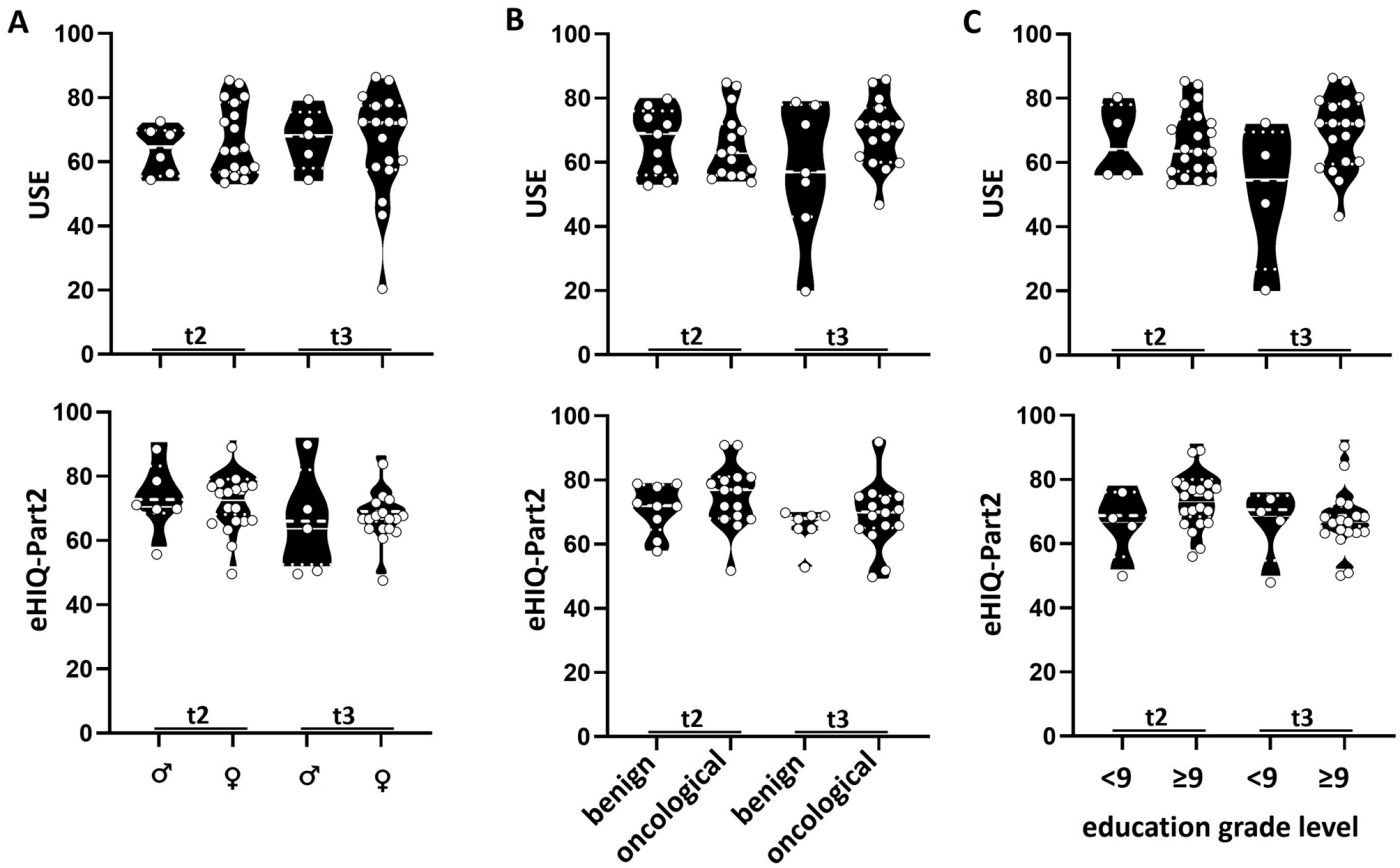
Scores for USE and eHIQ-Part2 before (t2) and after (t3) surgery according to (**A**) gender (male vs. female), (**B**) type of surgical indication (benign vs. oncological), and (**C**) education level (< 9th grade vs. ≥9th grade). The violin plots show individual patient data and indicate their respective medians and quartiles

Regarding perceived impact (eHIQ), patients’ attitudes toward the material were significantly positive before surgery (mean score 74 ± 9, above threshold), with somewhat lower but still favorable ratings after surgery (69 ± 10) (Fig. 2E). There were no differences in usefulness or impact according to patient characteristics (gender, type of surgical indication, and education level), acknowledging however the small size of some of the subgroups (Fig. 3). Among impact subscales, high scores in the “information and presentation” subscale highlighted the clarity, structure, and credibility of the content (Fig. 2F-H). Similarly, USE results were highest regarding the level of knowledge (cognitive subscale) compared to the other subscales (Fig. 1B-D). Finally, the lower overall Part 2 scores at t3 compared with t2 (Fig. 2E) seem to be due to the significantly lower scores in the “information and presentation” subscale (*p* = 0.0026) (Fig. 2G). This suggests that the perceived impact of the new information material decreases after surgery mainly because the perceived relevance of the procedure-related information is lower. The extent to which the information provided matched a particular patient’s actual lived experience may also play a role, at least for some patients.

There was a significant increase in general attitudes towards online health information (*p* = 0.033), with no significant difference in inclination towards sharing experiences with others online (Fig. 2C-D). This indicates that use of the new material increases patients’ trust on the internet as a source of health-related information, which is a very positive finding regarding the material’s impact. Conversely, the absence of an effect on inclination towards sharing experiences with others online is expected, because use of the material does not entail such interactions.

### Moderate-to-high stress, low anxiety and low depression before and after surgery

**Fig. 4:**
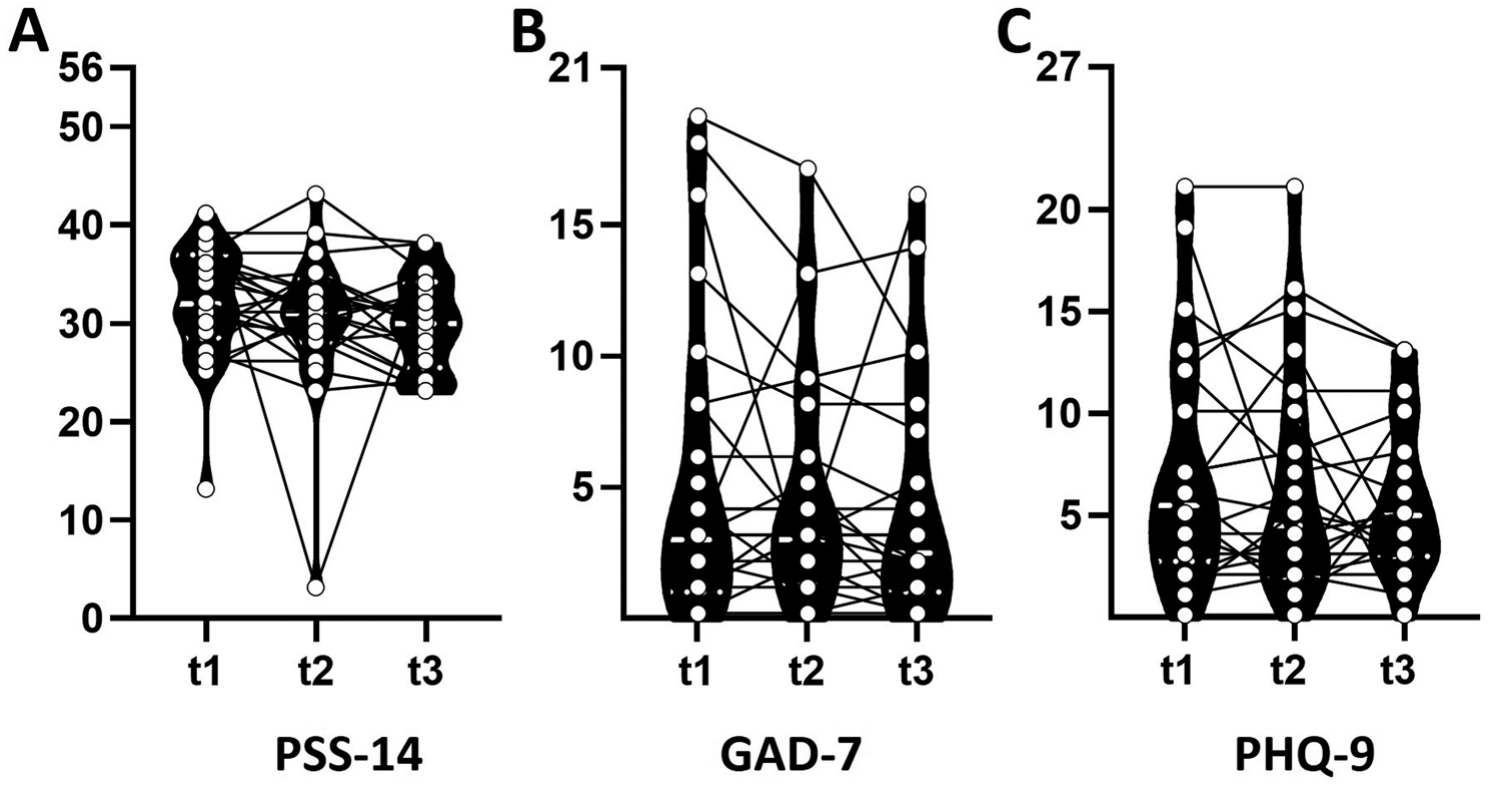
Scores for (**A**) Perceived Stress Scale-14 (PSS-14), (**B**) Generalized Anxiety Disorder-7 (GAD-7), and (**C**) Patient Health Questionnaire-9 (PHQ-9) before (t2) and after (t3) surgery. The violin plots show individual patient data and indicate their respective medians and quartiles

Stress levels were moderate to high throughout, with mean PSS-14 scores of 32 ± 6 at t1, 30 ± 7 at t2, and 30 ± 5 at t3 (Fig. 4A). Nearly all patients scored ≥ 19 (indicating at least moderate levels of perceived stress), with 2 at each time ≥ 38 (indicating high stress) [27].There were no significant differences in PSS-14 scores over time.

Anxiety levels varied considerably across patients, but mean GAD-7 scores remained stable over time: 5 ± 6 at t1, 5 ± 5 at t2, and 4 ± 5 at t3 (*p* = 0.73) (Fig. 4B), with only 3, 2, and 4 patients respectively scoring ≥ 10 (indicating at least moderate anxiety) [28]. The upward trend after access to the material and downward trend after surgery observed in some participants (Fig. 4B) might be related to the fact that these participants completed their questionnaire very close to the day of surgery. A participant with low level of anxiety before surgery that increased markedly after surgery (Fig. 4B) stated in the debriefing questionnaire that she did not experience what the information material described.

Depression also showed variability, though most patients had low symptom levels; mean PHQ-9 scores were 7 ± 6 at t1, 6 ± 5 at t2, and 6 ± 4 at t3 (Fig. 4C), with 5 patients at each time point scoring ≥ 10 (indicating at least moderate depression) [29]. Like PSS-14 and GAD-7 scores, there were no significant differences in PHQ-9 scores over time.

Baseline stress scores (PSS at t1) correlated positively with impact (eHIQ-Part 2) at t3 (*r* = 0.495, *p* = 0.019), whereas anxiety scores at t3 (GAD-7) correlated negatively with perceived usefulness (USE) at t3 (*r*=-0.713, *p* = 0.0002); no other correlations were observed between psychological parameters and impact or usefulness.

### Good content quality, understandability, actionability and linguistic suitability of the material

**Fig. 5:**
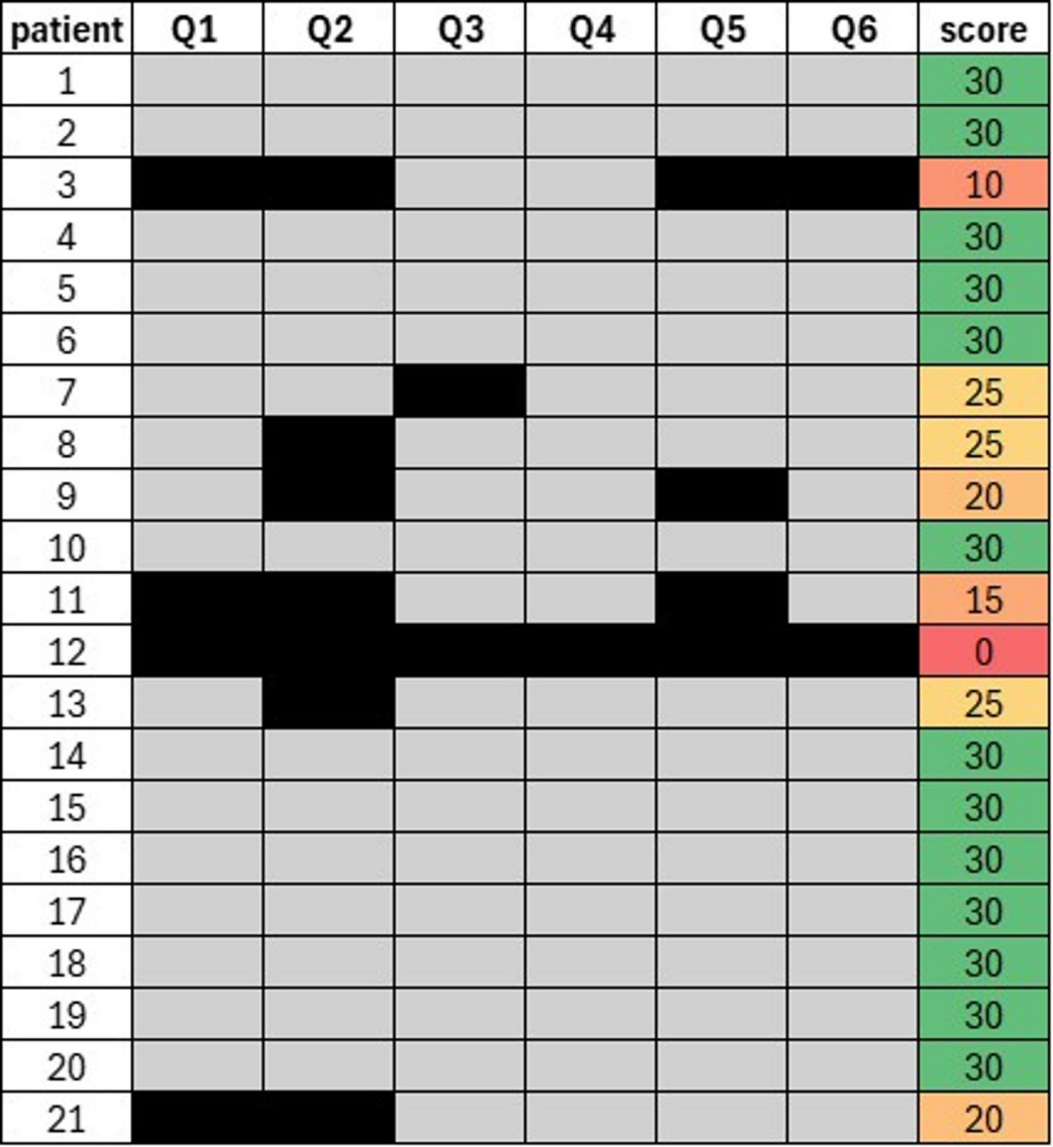
Individual patient responses and scores for the 6-question Brief DISCERN instrument, assessing the quality of the material’s content. “Yes” responses, awarded 5 points each, are shown in gray, and “no” responses, awarded 0 points each, are shown in black. Total scores are shown as a heatmap, with values ranging from 0 to 30

Quantitative and qualitative feedback via the custom debriefing questionnaire (Supplementary Table 1) indicated that most patients (87%) found the material useful or very useful, and none found it not at all useful. Patients reported that the material made them feel calm, relieved, confident, reflective and curious. All patients indicated trust in the source of the material and believed that the information provided is accurate and reliable. Their main topics of interest were thyroid function, what is going to happen, preparation for hospitalization, and the post-hospitalization period. No information was identified as upsetting or difficult to understand, and except for two patients, the information provided reportedly corresponded to what actually happened (Table 2). Patients’ responses in the validated Brief DISCERN tool [30] also indicated a very good content quality of the new audiovisual material. The majority (19/21, 90%) of patients who completed the instrument at the end of the study (t3) gave scores > 16, with 14/21 (67%) giving the maximum score of 30; the mean score was 25 ± 8 (Fig. 5).

**Table 2:** Results of the quantitative analysis of the debriefing questionnaire

	Aspects of the material	Example patient quote(s)
Support usefulness & impact	Clarity and conciseness	The content is good for informing about the disease.
	Relevance and applicability	Explanations of my case.
	Accessibility	The format is perfect.
	Empowerment	Useful to know the logical sequence of events to better prepare.
Could be improved	Limited scope	I was in stress to find information on exophthalmos, and I was disappointed not to find any. I would have liked to see more examples of recommended foods.
	Technical difficulties	It’s hard to read on a cell phone. I had to use a tablet. The font size is very small. The structure is well done but I sometimes got lost to find a page that I wanted to review.
	Language and cultural barriers	My suggestion is to subtitle the videos so that non-French speaking patients can understand better; then the text can be summarized.
	Contradictions with lived experience	At the hospital I was advised to take the calcium 2 h after the thyroid hormone and on the site, it is a 4-hour delay. I feel a little scared because the doctor had not explained all the details to me.

**Fig. 6:**
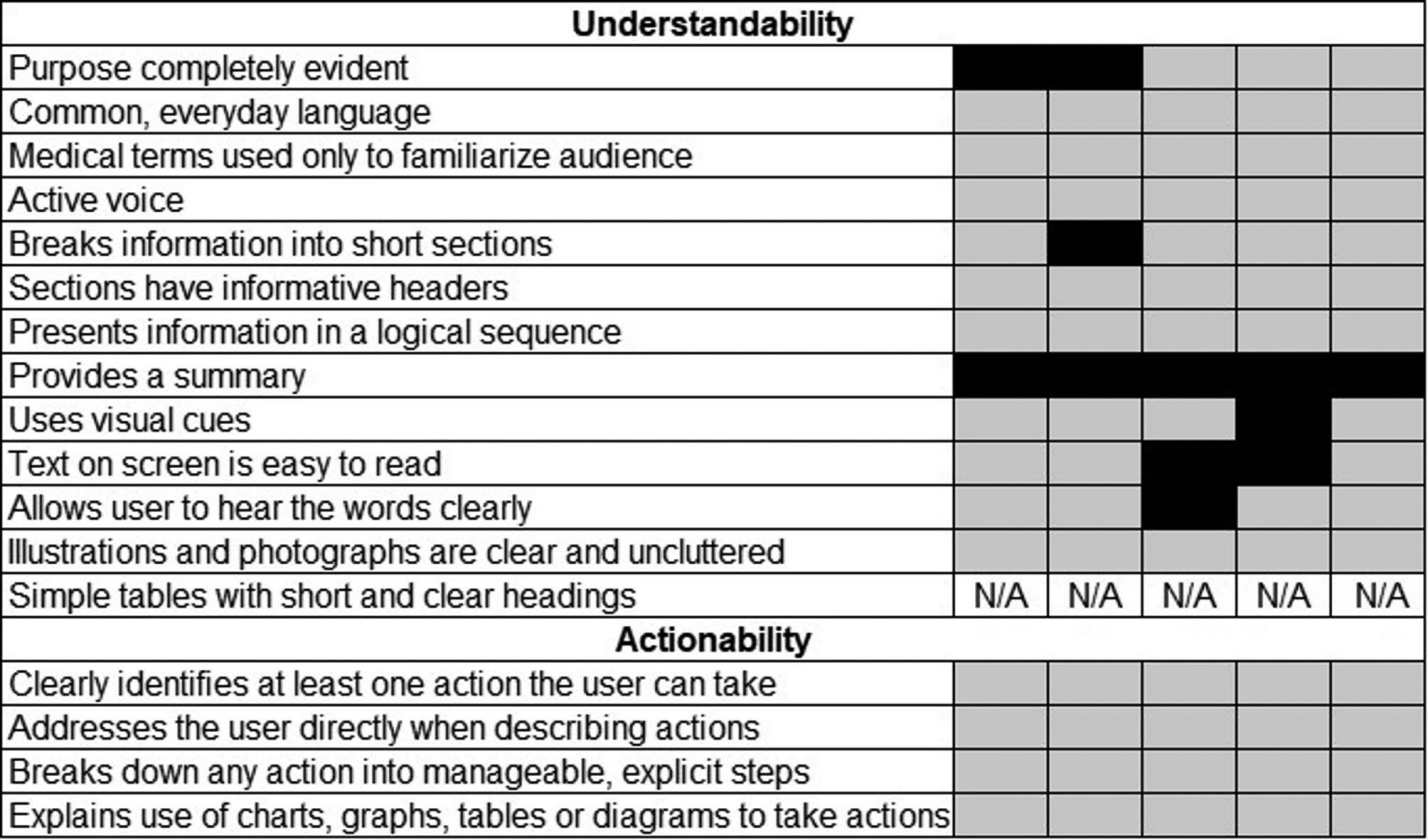
Individual investigator responses for Patient Education Materials Assessment Tool for Audiovisual Materials (PEMAT-A/V). To save space, the wording of some questions has been abbreviated, taking care to preserve their original meaning. The assessment itself was conducted using the original, validated PEMAT-A/V with the full wording of each question. “Yes” responses, awarded 1 point each, are shown in gray, and “no” responses, awarded 0 points each, are shown in black. N/A: not applicable

Using PEMAT-A/V, 5 investigators independently assessed the educational material’s understandability (13 questions) and actionability (4 questions), discussing and resolving uncertainties wherever relevant. Results showed a perfect actionability score of 100% and a very good understandability score of 82% (well above the 70% threshold) (Fig. 6). The inter-rater reliability, measured by Fleiss Kappa, indicated fair agreement among the 5 reviewers (κ = 0.39, *p* < 0.001). The material demonstrated strengths in understandability through well-structured information, effective visual aids, and use of everyday language; contextualization of the latter should consider that the investigators were HCPs.

Linguistic analysis using LIWC-22 [31] showed that the French text was longer and more lexically complex than the English version, though dictionary coverage was lower (73% vs. 87%). Valid LIWC measures for English indicated intermediate analytical style, high confidence (Clout = 84), lower authenticity, and a more negative tone. Emotion word use was higher in French (2.41% positive and 2.13% negative) than English (0.23% and 0.64%, respectively). Analysis of the material’s tone and style using YesChat Tone Analyzer [32] showed that the content effectively maintains a neutral and informative tone, using clear language to educate readers about thyroid function and related medical-surgical procedures in both versions, with English more conversational and French more formal (Table 3).

**Table 3:** Results of the tone style analysis of the text in the English and French versions

	English version	French version
Tone	Calm, reassuring and educational. Blends clinical accuracy with empathetic clarity.	Reassuring, instructive and compassionate. The text anticipates patient anxiety and responds with warmth and clarity.
Style	Formal yet accessible; uses structured headings, bullet points, and short paragraphs for readability.	Comprehensive and didactic, broken into structured headings and subheadings for ease of navigation. Uses bullet points, analogies and diagrams or placeholders to support explanation.
Voice	Neutral, informative, and professional. Consistently uses second person (“you”) to engage readers and personalize complex medical explanations.	Empathetic and authoritative. Speaks directly to the reader (“you”) in a professional yet human-centered manner.
Linguistic patterns	Definitions followed by examples or analogies; use of parenthetical clarification (e.g., “also known as T3”). Emphasis on demystifying technical terms. Gentle encouragement to ask questions, reduce anxiety, and participate in care. Softened clinical language (e.g., “not too painful,” “mild hoarseness”) to reduce fear.	Frequent use of parenthetical definitions (e.g., “T3 – triiodothyronine”). Mild repetition for reinforcement. Soothing language when referring to sensitive topics (e.g., cancer, surgery, biopsy). Present tense and active voice to maintain immediacy and support understanding. Occasional rhetorical questions to engage the reader (e.g., “What is an endocrinologist?”). Step-by-step procedural outlines to reduce uncertainty and build trust.

Finally, triangulation of results across content quality, readability and linguistic features, revealed trust in the source’s credibility, with the material being consistently assessed as clear, concise, and relevant.

### Areas for improvement in readability, understandability and navigability

Results of readability analysis by two investigators using standardized formulas (Table 4) indicated that both language versions were written at a higher complexity than the generally recommended 6th -8th grade level. Only one section in French and two in English met the recommendations. The greater complexity of most Sect. (14/18) in French compared to English is consistent with findings from previous evaluations of patient information materials [33, 34] and may be related to differences in language structure and/or their treatment by readability formulas [35].

**Table 4:** Readability analysis results per section of the English and French versions

Section	English version						French version							
	FKGL	GFI	SMOG	FRES	Grade	Reading level	FKGL	GFI	SMOG	FRES	Grade	Reading level	Scolarius	
1. Understand my condition	9.1	10.6	11.8	56.8	10th	somewhat difficult	11.9	14.6	13.8	40.1	12th	difficult	124	college
2. Understanding the diagnostic process	8.8	10.3	11.5	57.4	11th	fairly difficult	12.1	14.4	13.7	33.8	12th	difficult	139	college
3. Understand the treatment options	12.1	14.4	13.7	33.8	11th	fairly difficult	11.6	13.9	13.2	35.7	12th	difficult	120	college
4. Questions to ask to your surgeon	11.6	13.9	13.2	35.7	11th	fairly difficult	6.7	9.1	13.2	61.8	7th	average	57	primary
5. Understand the surgery - The thyroid	10.4	13.9	13.6	50.1	11th	fairly difficult	12.3	14.8	14.0	36.3	college entry	very difficult	138	college
6. What will happen before your surgery	11.2	14.1	13.3	50.3	11th	fairly difficult	14.3	16.5	15.2	28.0	12th	difficult	153	university
7. Medication advices	14.4	18.2	16.3	28.5	college	professional	16.0	18.7	16.4	15.1	college entry	very difficult	142	college
8. Understand my hospitalization	8.5	11.6	11.8	36.5	10th	somewhat difficult	11.9	14.4	13.5	38.6	11th	fairly difficult	115	secondary
9. What are the next steps after surgery	11.0	13.4	13.1	46.1	12th	difficult	13.0	15.6	14.4	32.3	12th	difficult	138	college
10. What to pack for my hospital	9.1	9.1	11.8	52.0	12th	difficult	11.3	13.9	13.4	38.4	11th	fairly difficult	168	university
11. How to manage anxiety	7.5	10.6	11.0	67.9	8th	average - slightly dif.	11.4	13.2	13.1	42.7	10th	somewhat dif.	174	university
12. Pre-admission check list	7.5	10.6	10.9	64.7	8th	average - slightly dif.	11.2	13.7	13.7	42.5	10th	somewhat dif.	329	initiated
13. Managing pain after your surgery	10.7	13.4	13.1	56.1	11th	fairly difficult	13.9	16.6	15.2	33.2	college entry	very difficult	159	university
14. Instruction for scar care after surgery	13.9	16.6	15.2	33.2	10th	somewhat difficult	13.8	16.3	15.2	34.2	12th	difficult	144	college
15. Managing your weight after surgery	9.1	11.3	11.7	58.7	11th	fairly difficult	11.8	14.5	13.9	39.9	12th	difficult	108	secondary
16. Recovery period recommendations	8.4	11.1	11.4	61.5	9th	slightly difficult	12.1	14.8	13.9	36.4	11th	fairly difficult	117	secondary
17. Hormone treatment	8.9	11.2	11.6	56.3	10th	somewhat difficult	12.1	14.4	13.8	35.8	11th	fairly difficult	157	university
18. Radioactive iodine treatment	10.7	13.2	12.8	47.9	12th	difficult	13.4	15.9	14.6	30.6	college entry	very difficult	134	college

The Flesch-Kincaid Grade Level (FKGL) indicates the U.S. grade level needed to understand the text, with lower scores indicating easier readability; the Gunning Fog Index (GFI) estimates the years of formal education needed to understand the text on first reading, with lower scores indicate easier readability; the Simple Measure of Gobbledygook (SMOG) estimates the years of education needed to understand a piece of writing, with lower scores indicating easier readability; the Flesch Reading Ease Score (FRES) scores text on a 100-point scale, with higher scores indicating easier readability; and Scolarius provides a comparison to other texts of the same type, with lower scores indicating easier readability

The investigators’ PEMAT-A/V assessment identified areas to maximize understandability. Specifically, a summary was lacking; some pages contained excessive text on a single page, reducing readability; illustrations could be better utilized to decrease text density; minor inconsistencies in terminology and occasional typographical errors were observed; topic separation and section transitions could be clearer; and some anatomy- and insurance-related terms would benefit from cultural adaptation for a Swiss audience.

Finally, open-ended patient feedback via the debriefing questionnaire highlighted mostly practical barriers with navigability (Table 2). Specifically, the small font size made the material difficult to use on smartphones, especially the videos; missing subtitles occasionally hindered understanding; and transitioning between sections, especially from the main menu, was not intuitive. Regarding the content, a few patients requested more comprehensive or specialized details such as nutritional advice or information on thyroid eye disease.

## Discussion

This study provides a first comprehensive evaluation of the Butterfly educational platform by Medtronic, Inc. designed for patients undergoing thyroid surgery. This type of evaluation is relevant because a study assessing patient information on thyroid cancer concluded that the quality of the respective websites is highly variable, with a predominance of low-quality content, highlighting the need for improved visibility of trustworthy sources and enhanced e-health literacy among patients [36]. Indeed, the information received has been identified as an area for improvement even among thyroid cancer patients who were generally satisfied [37]. Further, an analysis of unmet information needs among thyroid cancer survivors showed that a significant proportion reported insufficient understanding before treatment [38]. Such findings indicate that, even though online resources cannot replace the information provided orally by HCPs, ensuring their quality, content and accessibility is essential to support informed decision-making and improve health outcomes.

### Strengths of the material and of the study

In contrast to other online resources that often lack consistency, accuracy, and/or clinical applicability, the Butterfly platform combines a positive patient experience with a content validated by HCPs, making for a credible preoperative educational tool. Of note, the platform was perceived as trustworthy, appropriate, and of good quality even by participants reporting only moderate reliance on online health information. Participants particularly appreciated the clarity and reliability of the written materials, while HCPs confirmed its clinical applicability, as evidenced by perfect actionability and good understandability ratings. Importantly, the surgical information provided aligned closely with almost all patients’ actual experiences. Furthermore, text analysis using informatics tools demonstrated a neutral and informative tone, characterized by clear, balanced, accessible, and empathetic language, well-suited to educating and reassuring patients.

The inclusion of audiovisual content represents another strength, as videos were systematically viewed and appreciated, aligning with evidence that patients learn effectively from visual material. In general, young adults find the video format easy to learn from [39]. In addition to digital and health literacy, concerns about privacy represent barriers to the adoption of digital health technology [40]; no such concerns were raised by the participants, which was reassuring. Other critical factors influencing digital health engagement, such as comprehensibility, visual appeal, credibility, and adaptability [41], were also judged positively, with minor usability issues identified for improvement.

A key strength of this study is the use of multiple tools, both quantitative and qualitative, to evaluate various relevant aspects of the new educational material. In addition to achieving a comprehensive assessment, this also facilitated the cross-validation of the main findings; for example, the high understandability and actionability indicated by the investigators in PEMAT-A/V mirrored the high level of understanding and motivation indicated by the patients in eHIQ Part 2. Similarly, there was clear correspondence between patients’ responses in USE, eHIQ Part 2 and the qualitative debriefing questionnaire, as well as between these three questionnaires and the automated results of the linguistic and tone analysis by informatics tools on respective aspects. These consistencies among the assessment results by patients (providing genuine insights and feedback about their experiences), researchers (maintaining scientific rigor in the methodology) and informatics tools (avoiding bias) strongly support the internal validity of the present evaluation, as does the overall stability of most responses across the respective time points.

### Study limitations

One limitation is the small sample size, with 22/26 patients completing the study (drop out-rate of 15%; one died from an aggressive brain cancer diagnosed shorty after t2; one was managed in another center after t1; one postponed the surgery after t1 until after the study, and the last one dropped out after t2 for unclear reasons). The study was completed by 22 patients, below the initially calculated target of 27. This reduced sample size limited the statistical power particularly regarding subgroup comparisons. Nevertheless, the quantitative analysis of the results for the main outcome was statistically significant, and in the qualitative analysis saturation was reached with the available responses. The acceptance rate was high (60%), and participants who completed the study showed strong motivation and engagement throughout. We did not find differences in the main outcome according to gender, type of surgical indication or educational level, but the study was not specifically designed or powered to detect such differences. Finally, patient access to the material was not monitored by the platform, and whether patients consulted the material again especially after surgery was not asked. Notwithstanding this limitation, the specific study design corresponded well to real-life practice, where patients are given access to resources and are free to consult them as they wish.

In addition to the small sample size, generalizability of the results may be limited due to the fac that the cohort was recruited from a single Swiss institution and had relatively high health literacy. Thus, the transferability of the findings to more diverse populations, such as those with different cultural or linguistic backgrounds, lower health literacy, or restricted access to digital resources remains to be examined. This is mainly evident in the readability results, where standardized metrics indicated text complexity, yet participants reported no difficulties in comprehension, likely thanks to the higher educational level of the study population. Finally, participants continued to report elevated stress levels throughout the study, indicating that although the platform improves knowledge and provides reassurance, it is not sufficient as a stand-alone intervention to alleviate the psychological burden associated with impending surgery and the post-surgical period. Evidence from other studies, such as randomized controlled trials, shows that nurse-led web-based patient education can significantly reduce anxiety in patients scheduled for thyroid surgery and may improve early postoperative recovery [42]. Beyond thyroid surgery, findings from recent umbrella reviews [43] and other studies conclude that digital educational tools can help to reduce psychological symptoms such as stress, anxiety and depression [44–46]. Notably, a recent meta-review of 31 meta-analyses (encompassing 505 studies) found that human-supported digital mental health interventions are more effective than unsupported ones, especially for individuals with more severe symptoms [47]. Taken together, the above findings suggest that, in parallel with using Medtronic’s Butterfly platform to educate patients with indications for thyroid surgery, HCPs could consider screening these patients for stress and offering appropriate support as needed.

### Complementarity with decision aids in shared decision-making

Recent literature highlights the importance of both specialized decision aids and robust patient education in supporting informed decision-making across the thyroid care continuum. For instance, Singh Ospina and colleagues developed and pilot-tested a web-based conversation aid specifically designed to facilitate structured discussions on thyroid nodule evaluation, demonstrating improved patient activation, clinician satisfaction, shared understanding, and reduced unnecessary biopsy procedures through enhanced discussions of management options [48]. Similarly, Melcarne and colleagues implemented a SPIKES (Settings, Perception, Invitation, Knowledge, EmotionS)-based protocol in an Italian cohort to communicate diagnostic uncertainty in indeterminate thyroid cytology, using a mixed-methods approach to refine communication delivery and strengthen decisional quality prior to surgical referral [49]. These examples demonstrate the value of structured approaches to patient communication and decision-making. The Butterfly platform operates in a complementary space, functioning as a comprehensive audiovisual educational resource rather than a treatment decision aid. By providing patients with clear, trustworthy information about their thyroid conditions, diagnostic processes, surgical procedures, and postoperative recovery expectations – applicable across the full spectrum of surgical indications from benign diseases and Graves’ disease to malignant conditions – the platform establishes the diagnostic understanding and procedural clarity that enables patients to engage meaningfully in shared decision-making discussions with their healthcare providers, including the use of decision aids like those mentioned above. This educational foundation aligns with the patient-centered principles emphasized in recent guidelines, such as the 2025 American Thyroid Association (ATA) guidelines on differentiated thyroid cancer management, which highlight the importance of informed patient participation in clinical decision-making [50].

## Conclusions

These findings provide preliminary support for the Butterfly platform as a useful educational resource in thyroid surgery. In our center, after the initial in-person consultation for thyroid surgery candidates, we direct patients to use the platform for detailed information and reference. We make sure that the surgeons responsible for their care are fully informed about the specific content patients have access to, so they can effectively address any questions the patients raise based on that information. Findings from this single-center study should be confirmed in larger and more diverse populations to fully establish the platform’s utility for patients with indication for thyroid surgery.

## Electronic Supplementary Material

Below is the link to the electronic supplementary material.

Supplementary Material 1

## Data Availability

No datasets were generated or analysed during the current study.
