# Supplementary material for: Comprehensive evaluation of Medtronic’s Butterfly platform, a new audiovisual information material for patient education and shared decision-making in surgical thyroid disease
Source: BMC Med Inform Decis Mak. 2026 Apr 29;26:219. doi: 10.1186/s12911-026-03526-w (PMC13270795; doi:10.1186/s12911-026-03526-w)
Supplement: Supplementary file 1 — Supplementary Material 1 [file 12911_2026_3526_MOESM1_ESM.docx]

**Project Butterfly – Debriefing Questionnaire**

| Patient ID: | Interview Date: (dd/mm/yyyy) |
| --- | --- |

**Regarding the informational material as a whole**

1. Overall, did you find the material useful for you?
   0 = Not at all 1 = A little 2 = Fairly 3 = Very 4 = I did not consult the material
   If yes, what was the most useful part for you?
   ...............................................................................................................................
2. What emotions did the use of the material trigger in you? (e.g., satisfaction, reflection, courage, fear, confidence, fatigue, surprise, confusion, calm, anxiety, relief, doubt, etc.)
   ...............................................................................................................................
3. Were there any pieces of information that you found confusing or difficult to understand?
   0 = No 1 = Yes
   If yes: Please specify below the information you found confusing or difficult to understand
   ...............................................................................................................................
4. Have you acquired new knowledge about your illness?
   0 = Not at all 1 = A little 2 = Fairly 3 = A lot
5. Do the information provided in the material correspond well to what actually happened?
   0 = No 1 = Yes
   If no: Please indicate below what was discrepant between the information provided and your experience
   ...............................................................................................................................
6. Were there any pieces of information that you found upsetting?
   0 = No 1 = Yes
   If yes: Please specify below the information you found upsetting
   ...............................................................................................................................
7. Would you recommend this material to other patients?
   0 = No 1 = Rather no 2 = Rather yes 4 = Yes
8. Have you accessed other information materials to obtain relevant information about your thyroid disease?
   0 = No 1 = Yes
   If yes, please specify which one(s)
   ...............................................................................................................................
9. Do you have any other comments about the information material?
   ...............................................................................................................................

**Regarding the web pages**

1. Did you consult the web pages?
   0 = Not at all 1 = A little 2 = Fairly 3 = A lot
2. Did you find the content of the web pages satisfactory?
   0 = Not at all 1 = A little 2 = Fairly 3 = A lot 4 = I did not consult the web pages
3. Do you have comments on the content of the web pages?
   ...............................................................................................................................
4. Did you find the format of the web pages satisfactory?
   0 = Not at all 1 = A little 2 = Fairly 3 = A lot 4 = I did not consult the web pages
5. Do you have comments on the format of the web pages?
   ...............................................................................................................................

**Regarding the videos**

1. Did you consult the videos?
   0 = Not at all 1 = A little 2 = Fairly 3 = A lot
2. Did you find the content of the videos satisfactory?
   0 = Not at all 1 = A little 2 = Fairly 3 = A lot 4 = I did not consult the videos
3. Do you have comments on the content of the videos?
   ...............................................................................................................................
4. Did you find the format of the videos satisfactory?
   0 = Not at all 1 = A little 2 = Fairly 3 = A lot 4 = I did not consult the videos
5. Do you have comments on the format of the videos?
   ...............................................................................................................................

**Regarding the forms and memo sheets**

1. Did you use the forms and/or the memo sheets?
   0 = Not at all 1 = A little 2 = Fairly 3 = A lot
2. Did you find the content of the forms and memo sheets satisfactory?
   0 = Not at all 1 = A little 2 = Fairly 3 = A lot 4 = I did not consult the forms and memo sheets
3. Do you have comments on the content of the forms and memo sheets?
   ...............................................................................................................................
4. Did you find the format of the forms and memo sheets satisfactory?
   0 = Not at all 1 = A little 2 = Fairly 3 = A lot 4 = I did not consult the forms and memo sheets
5. Do you have comments on the format of the forms and memo sheets?
   ...............................................................................................................................

**Regarding the questionnaires**

1. Were there any questions in the different questionnaires that you found confusing or difficult to answer?
   0 = No 1 = Yes
   If yes: Please note below the question number(s) you found confusing or difficult to answer
   ...............................................................................................................................
2. Were there any questions in the different questionnaires that you found upsetting?
   0 = No 1 = Yes
   If yes: Please note below the question number(s) you found upsetting
   ...............................................................................................................................
3. Do you have any other comments on the questionnaires?
   ...............................................................................................................................
